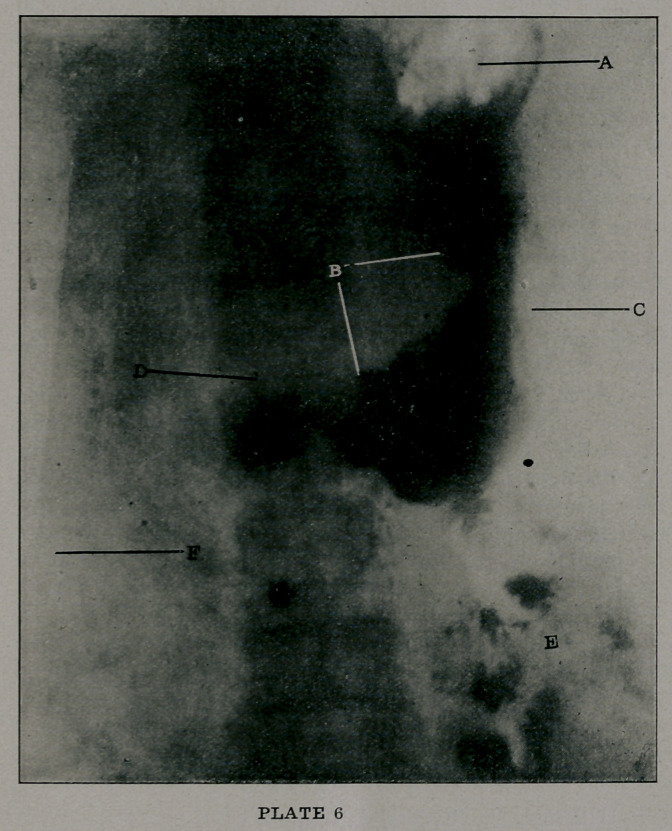# Six Roetgenograms Illustrating Some Commoner Gastro-Intestinal Disorders

**Published:** 1914-05

**Authors:** George M. Niles

**Affiliations:** Atlanta, Ga.


### Journal-Record of Medicine

Successor to Atlanta Medical and Surgical Journal, Established 1855
and Southern Medical Record, Established 1870

**OWNED BY THE ATLANTA MEDICAL JOURNAL COMPANY**

PUBLISHED MONTHLY

*Official Organ Fulton County Medical Society, State Examining Board, Presbyterian Hospital, Atlanta, Birmingham and Atlantic Railroad Surgeons' Association, Chattahoochee Valley Medical and Surgical Association, Etc.*

EDGAR BALLENGER, M. D., Editor
BERNARD WOLFF, M. D., Supervising Editor
A. W. STIRLING, M. D., C. M., D. P. H.; J. S. HURT, B. Ph., M.D.
GEO. M. NILES, M. D., W. J. LOVE, M. D., (Ala.); Associate Editors
E. W. ALLEN, Business Manager

**COLLABORATORS**

DR. W. F. WESTMORLAND, General Surgery
F. W. McRAE, M. D., Abdominal Surgery
H. F. HARRIS, M. D., Pathology and Bacteriology
E. B. BLOCK, M. D., Diseases of the Nervous System
MICHAEL HOKE, M. D., Orthopedic Surgery
CYRUS W. STRICKLER, M. D., Legal Medicine and Medical Legislation
E. C. DAVIS, A. B., M. D., Obstetrics
E. G. JONES, A. B., M. D., Gynecology
R. T. DORSEY, Jr., B. S., M. D., Medicine
L. M. GAINES, A. B., M. D., Internal Medicine
GEO. C. MIZELL, M. D., Diseases of the Stomach and Intestines
L. B. CLARKE, M. D., Pediatrics
EDGAR PAULIN, M. D., Opsonic Medicine
THEODORE TOEPEL, M. D., Mechano Therapy
R. R. DALY, M. D., Medical Society
A. W. STIRLING, M. D., Etc., Diseases of the Eye, Ear, Nose and Throat
BERNARD WOLFF, M. D., Diseases of the Skin
E. G. BALLENGER, M. D., Diseases of the Genito-Urinary Organs

VOL. LXI ATLANTA, GA., MAY, 1914. NO 2

### SIX ROETGENOGRAMS ILLUSTRATING SOME COMMONER GASTRO-INTESTINAL DISORDERS.

GEORGE M. NILES, M. D., ATLANTA, GA.

The importance of roentgenography in the diagnosis of gastro-intestinal disorders, organic and otherwise, has passed the debatable stage, and become a recognized entity. Furthermore, the aid to be obtained in clearing up obscure conditions is now admitted not only by the members of the medical profession, but is becoming well known to the laity. A short time ago I was much surprised to hear a kinky-headed, ebon-hued son of Ham at my clinic make the request that I "look through him" so I could tell him what was his trouble.

PLATE 1

Now that the X-ray tidings are being disseminated among all walks in life, so that even the ignorant and benighted members of society beseech us for its assistance, it behooves us to carefully heed this subject, so that our patients may not have the shadow of an excuse for considering us derelict in all proper diagnostic efforts.

I wish to present herewith several roentgenograms, each one of which conveys its lesson. Some have gone to operation, with confirmation of the X-ray findings; some have not. In every instance the roentgenographic findings have afforded positive in-

PLATE 2

formation of either practical or theoretical character, so that if no tangible benefit followed, at least a definite opinion could be advanced.

Plate 1 shows a chronic, non-indurated gastric ulcer, with the lesion in the posterior wall of the pars pylorica, as shown by B. A shows the outline of the stomach, C the pylorus, D the pyloric cap, or better termed, the pars duodenale. The cecum and ascending colon are also shown at E.

This case came to operation, and the diagnosis was confirmed.

PLATE 3

In Plate 2, can be seen characteristic signs of hypermotility of the stomach, associated with a moderate degree of gastroptosis. The blurred image of the fundus at A betokens an accumulation of gas, while B and B show rugae in the walls of the viscus. The pyloric contraction is easily visible at D, and a normal duodenal cap can be observed at E. F shows the third portion of the duodenum, and the liver shadow can be dimly discerned at G.

**PLATE 4**

This is a most instructive roentgenogram, portraying a disturbed state of the stomach accompaying either pylorospasm, slight stricture of the pylorus, or even an emotional disorder. In this particular instance, the trouble seemed purely a neurosis.

Plate 3 illustrates a rather extreme condition brought about by severe and lengthy pylorospasm. In this we see at B and B a stomach enlarged transversely and longitudinally, gas in the fundus at A, a very tight pylorus at C, and the first part of the duodenum at D. In this patient there was a considerable residue left in the stomach after nineteen hours, while a general sense of abdominal distress with frequent regurgitations of ill-smelling and ill-tasting food and gas increased the discomfort.

**PLATE 5**

Plate 4 shows a typic "water-trap" stomach, which is a gross exaggeration of the normal "fish-hook" organ, as found in many tall and slender individuals. In this type there are dragging pains, disinclination to lift heavy burdens, poor drainage from the stomach, and much gastric flatulence. These patients are generally ill-nourished, pessimistic, and "dyspeptic" in the popular acceptation of the term. Such conditions, however, lend themselves kindly to surgical intervention, and I have seen highly satisfactory results follow well-directed operative efforts in several instances under my own observation.

The greatly elongated "arm" of the stomach may be observed at A, while the acute angle of the lesser curvature is graphically depicted at B.

PLATE 6

Plate 5 illustrates a well-marked coloptosis as well as several other conditions of interest both to the physician and patient. The dilated and prolapsed cecum is noted at A, a prolapsed hepatic flexure at C, a diseased appendix, showing a short and irregular lumen at B, adhesions at D, the prolapsed transverse colon at E, prolapsed splenic flexure at F, and the sigmoid at G.

This condition is quite frequent in multiparous women, who have not been well cared for, and in elderly people of both sexes, who have lapsed from a robust and well-nourished state to a feeble and flaccid physique. Many of these are constipated to an extereme degree , are "pot-bellied" just above the symphysis are spare water drinkers, and lead a miserable existence.

Plate 6 tells the melancholy tale of gastric carcinoma. A shows a collection of gas in the fundus, B a marked involvement by new growth of the lesser curvature and pars pylorica. C shows probable metastases in the outer part of the body of the stomach, D the duodenum, E flecks of bismuth in the small intestines, and F, gas in the splenic flexure of the colon. Cases this far advanced are seldom amenable to operation, and the one here exhibited was no exception.

When a malignant growth has become extensive, with a palpable tumor, a roentgenogram only serves to prove what physical and clinical findings have already forecasted.

Roentgenology is no fad—it is here to stay, and it merits the thoughtful consideration of every earnest student of medicine.

920-22 Candler Building.

## Figures and Tables

**PLATE 1 f1:**
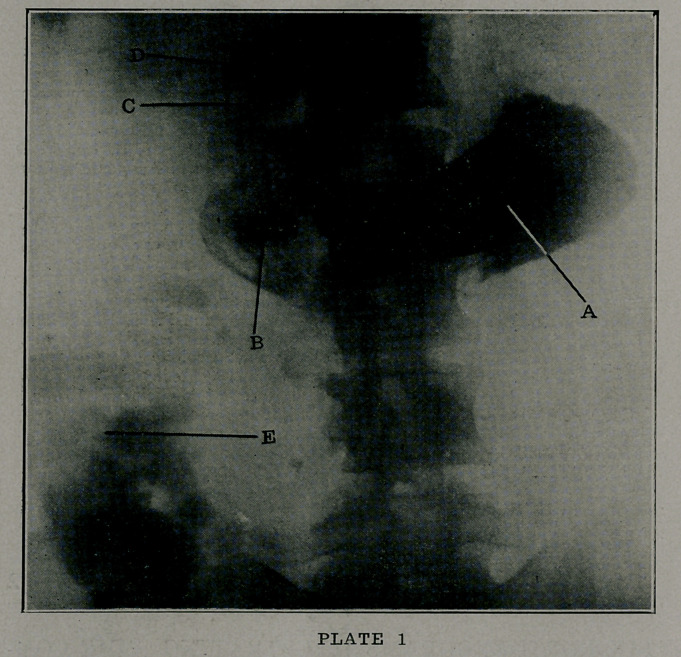


**PLATE 2 f2:**
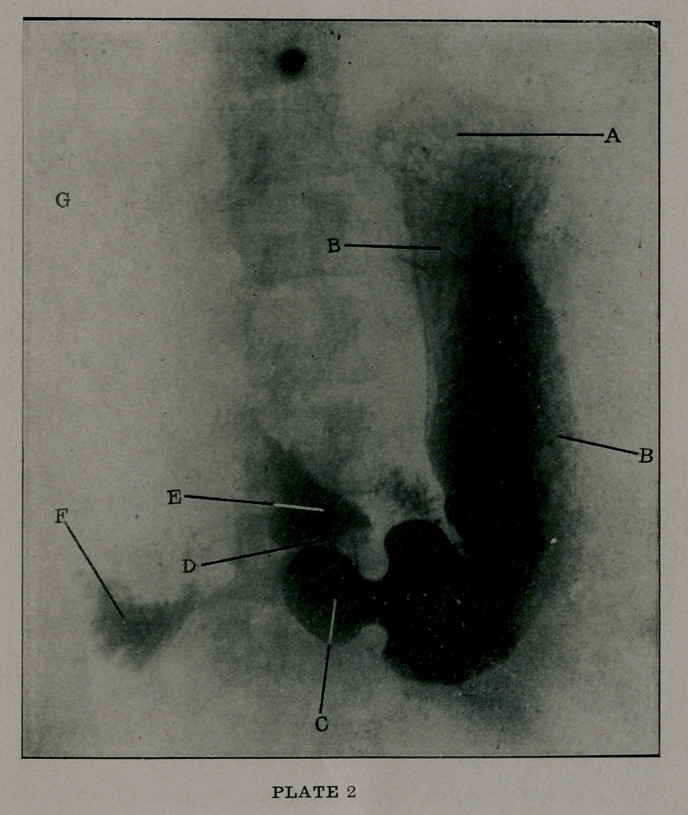


**PLATE 3 f3:**
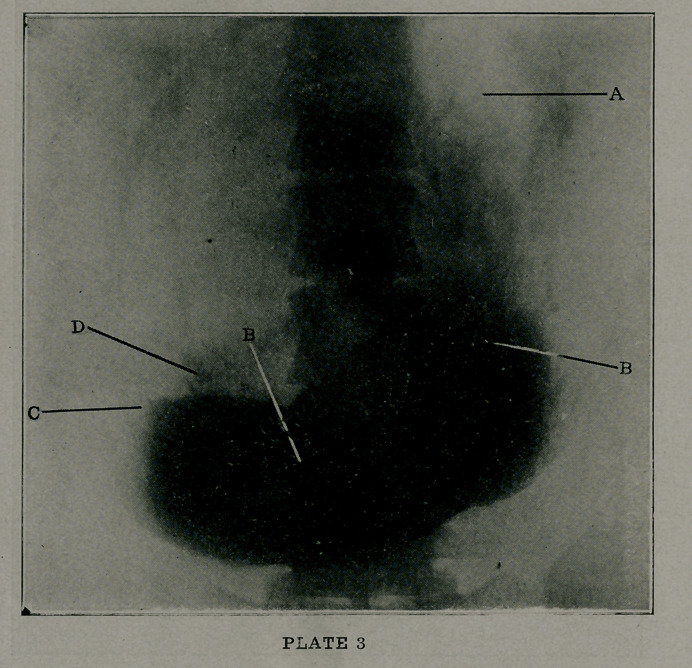


**PLATE 4 f4:**
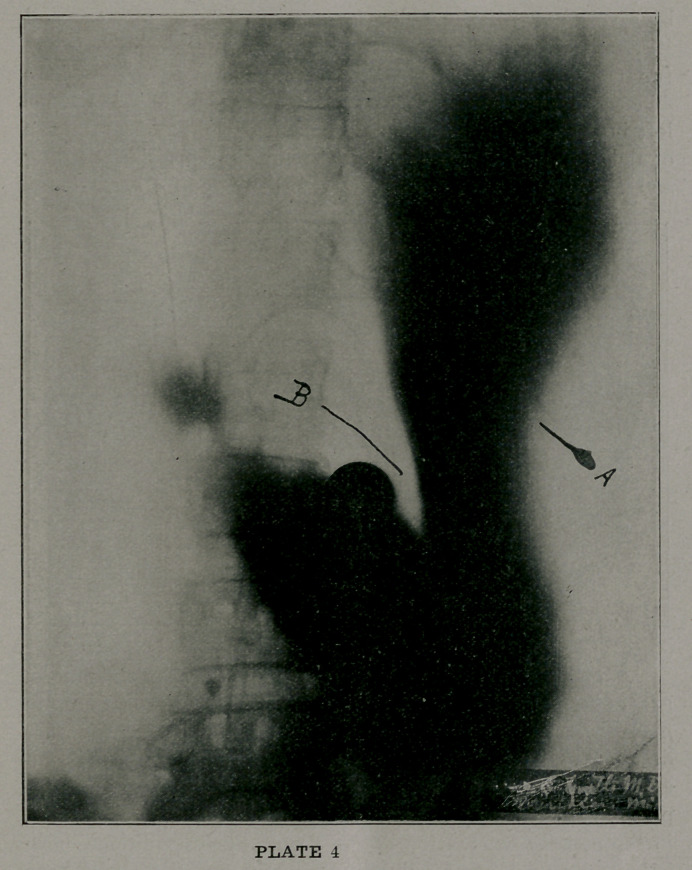


**PLATE 5 f5:**
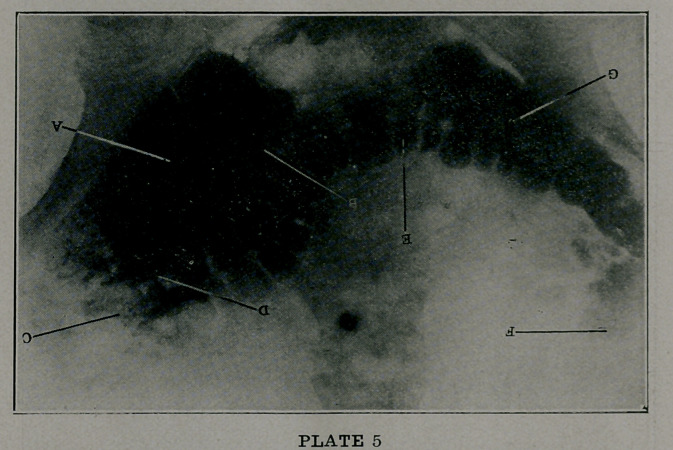


**PLATE 6 f6:**